# Cholesterol in viral envelope determines infectivity of SARS-CoV-2 and other coronaviruses

**DOI:** 10.3389/fmicb.2025.1670356

**Published:** 2025-09-18

**Authors:** Xiaoying Xu, Xueyan Wang, Xiafeng Zhang, Xin Jin, Yun Tan, Lin Huang, Mingqian Zhou, Chengping Wen

**Affiliations:** ^1^College of Basic Medical Sciences, Zhejiang Chinese Medical University, Hangzhou, China; ^2^School of Life Sciences, Zhejiang Chinese Medical University, Hangzhou, China

**Keywords:** human coronaviruses, viral envelope cholesterol, entry efficiency, viral attachment, therapeutic target

## Abstract

The SARS-CoV-2 pandemic had unprecedented impacts on public health and the economy. Many studies have focused on the mechanisms of SARS-CoV-2 entry into host cells, particularly the spike (S) protein mediated receptor engagement and subsequent virus-host membrane fusion dynamics. However, the mechanistic contribution of cholesterol within spike-incorporated viral envelopes to infectivity has not been well characterized. Herein, we show that targeted cholesterol depletion from the viral envelopes of SARS-CoV-2, SARS-CoV, and MERS-CoV directly impaired viral infectivity in a dose-dependent manner. Although modulation of host cell membrane cholesterol exerted relatively minor effects on viral entry, host cellular cholesterol homeostasis critically governs progeny virion infectivity by determining cholesterol content within nascent viral envelopes. Virions derived from cells with reduced plasma membrane cholesterol demonstrate significantly attenuated infectivity in SARS-CoV-2 and related coronaviruses. In addition, we detected that exogenous cholesterol replenishment restored SARS-CoV-2 entry efficiency by augmenting virus attachment. Collectively, our data demonstrate that biophysical properties of human coronavirus envelopes, particularly cholesterol stoichiometry, function as a key molecular determinant regulating host cell susceptibility. These findings position viral lipidome remodeling as a viable therapeutic target for developing host-directed broad-spectrum antivirals.

## Introduction

Coronaviruses represent a phylogenetically diverse family of enveloped viruses containing the largest known RNA genomes, characterized by positive-sense single-stranded RNA architecture. SARS-CoV-2, a novel *Betacoronavirus* clade member, shares evolutionary lineage with pandemic predecessors SARS-CoV (2002, China) and MERS-CoV (2012, Arabia) (Peng et al., 2021). Following the initial emergence of SARS-CoV-2-associated COVID-19 in December 2019, genetically distinct viral variants have persistently circulated worldwide through enhanced transmissibility and immune evasion (Zhang et al., 2024). It is urgent to develop cross-protective countermeasures against these highly pathogenic human coronaviruses.

The initial step of coronavirus infection involves the specific binding of the S protein to the cellular entry receptors. Association of the two proteins underlies virus-cell adhesion, which then induces the conformational change of S protein that bring the viral and cellular membranes together, ultimately creating a fusion pore through which viral genome is released into the host cytoplasm (Jackson et al., 2022). Given the membrane-dependent nature of these processes, cholesterol in the target cell membrane emerges as an essential determinant in mediating these pathophysiological events (Casari et al., 2021). Previous studies have demonstrated the critical role of cellular membrane cholesterol in viral infectivity, where uptake of cholesterol into cell membrane renders cells highly susceptible to infection. In contrast, depletion of accessible cholesterol on plasma membrane directly restricts SARS-CoV-2 infection (Zang et al., 2020; Wang et al., 2020).

In addition to facilitating SARS-CoV-2 entry into target cells, cholesterol-enriched membrane microdomains of the host cell are also critically required for the assembly of progeny virions. Following the first replication cycle, the establishment of systemic infection necessitates mass production of progeny virions. Given the absence of endogenous membrane biosynthesis pathways, nascent virions acquire lipid bilayer from their host cell to assemble its lipid envelope (Mesquita et al., 2021). Targeting this mechanism, SARS-CoV-2 infection hijacks host cholesterol trafficking to remodel membrane cholesterol levels, thereby determining the cholesterol content in the envelopes of newly budded virions (Doyle et al., 2024; La Rosa et al., 2024; Wang et al., 2023). However, the functional implications of cholesterol within SARS-CoV-2 envelope have received less attention. Therefore, we developed a VSV-based pseudovirus system, combined with envelope cholesterol modulation, to assess the impact of virion-associated cholesterol on SARS-CoV-2 infectivity in Vero E6 and Caco-2 cells, revealing a positive correlation between envelope cholesterol levels and viral infectivity. Our data provide a mechanistic basis for developing lipid-targeting entry inhibitors with pan-coronavirus efficacy.

## Results

### 25-hydroxycholesterol treatment shows no significant inhibitory effect on the entry of human coronaviruses *in vitro*

25-hydroxycholesterol (25-HC) is the product of cholesterol hydroxylation catalyzed by the enzyme cholesterol-25-hydroxylase (CH25H). 25-HC has been demonstrated to exhibit broad inhibitory activities against enveloped viruses of different families (Blanc et al., 2013; Zhang et al., 2023). 25-HC is upregulated in COVID-19-infected patients, and exerts anti-SARS-CoV-2 activity *in vivo* (Wang et al., 2020; Zu et al., 2020). Given the established role of 25-HC in selectively depleting accessible cholesterol from host cell plasma membranes, we utilized this compound to investigate whether target cell membrane cholesterol affects viral entry.

**Figure 1:**
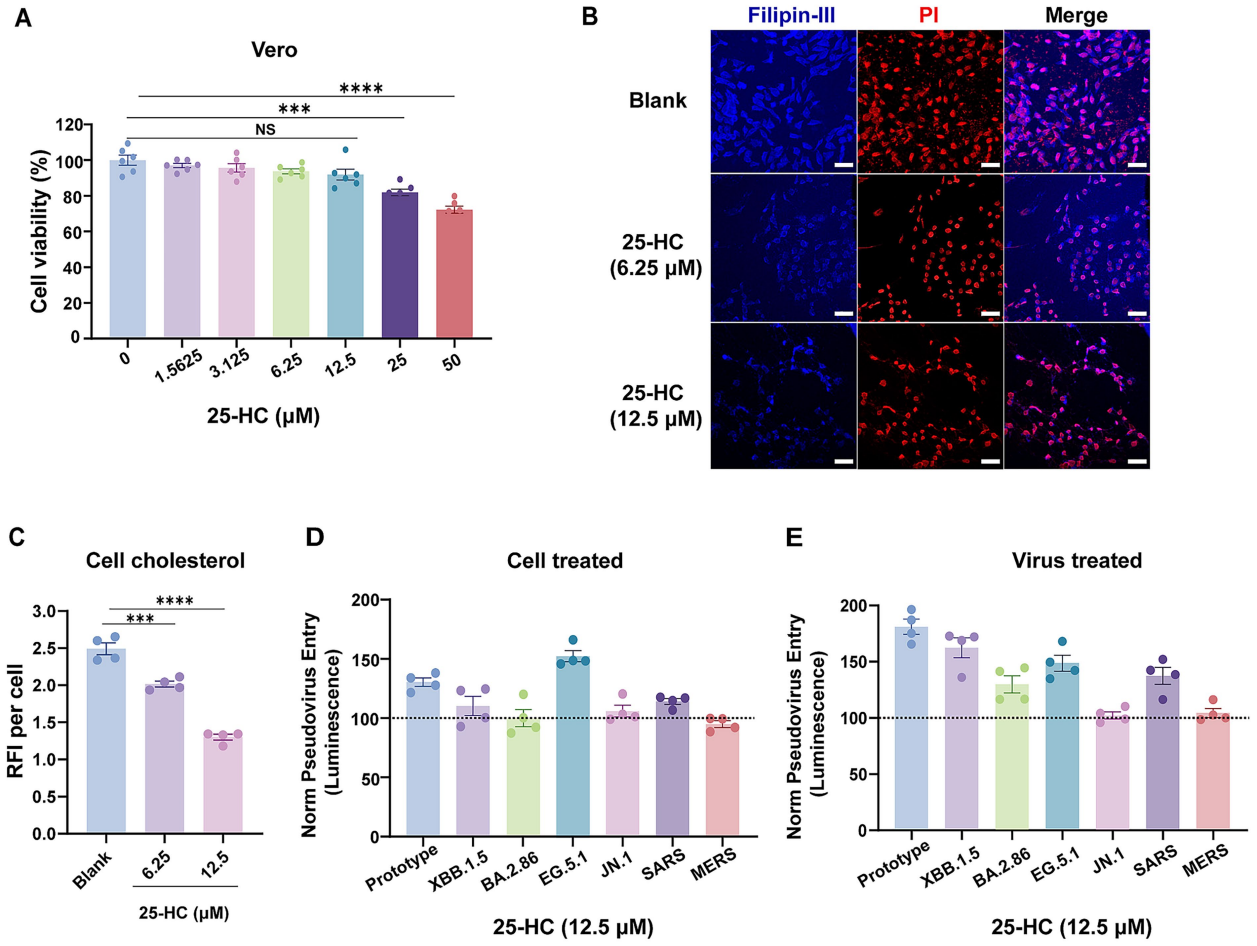
25-hydroxycholesterol treatment shows no significant inhibitory effect on the entry of human coronaviruses *in vitro*. **(A)** Cytotoxicity of 25-HC to Vero E6 cells was measured by CCK-8 assays. The Y-axis values of the graph represent the mean percentage of viable cells relative to untreated controls. **(B,C)** Immunofluorescence analysis of cholesterol levels in Vero E6 cells that received 25-HC treatment. Cholesterol was labeled with filipin-III, and the nuclei were stained with Propidium Iodide. The cholesterol distribution was measured by confocal microscopy and quantified by ImageJ. Scale bars, 50 μm. **(D,E)** 25-HC does not inhibit entry of SARS-CoV-2 pseudovirus, SARS-CoV pseudovirus, and MERS-CoV pseudovirus. Vero E6 cells were pre-treated with 25-HC for 24 h, and then infected with pseudotyped viruses expressing spike proteins from SARS-CoV-2 and its variants, as well as SARS-CoV and MERS-CoV. After 24 h, luciferase activity in cell lysates was measured and compared with the ethanol-treated control groups to quantify viral infectivity **(D)**. Panel **(E)** Similar to **(D)**, but pseudovirus were pre-treated with 25-HC, and viral entry efficiency was assessed by luciferase assay at 24 h post-infection. All data are shown as means ± SEM, *NS* not significant, ***p < 0.001, ****p < 0.0001. Statistical significance was determined by two-tailed unpaired Student’s *t* test for two groups or one-way ANOVA with Tukey’s HSD test for multiple groups.

We first tested the cytotoxicity of 25-HC in Vero cells by standard CCK-8 assay. 25-HC had no obvious cytotoxicity at concentration as high as 12.5 μM (Figure 1A). To confirm the ability of 25-HC to modulate the cholesterol on the plasma membrane, filipin-III, a fluorescent probe for cholesterol, was used to visualize the cholesterol of Vero E6 cells after 25-HC treatment. Confocal laser microscopy demonstrated significantly higher cholesterol levels in untreated cells relative to 25-HC-treated counterparts. Furthermore, the fluorescence signal decreased progressively with higher 25-HC concentrations indicated that 25-HC depleted plasma membrane cholesterol in a dose-dependent manner (Figures 1B,C). We then employed VSV-based pseudotyped particles incorporating spike proteins from SARS-CoV-2 prototype strain, emerging variants, and two other human coronaviruses, SARS-CoV and MERS-CoV to investigate the direct regulatory effects of target cell membrane cholesterol on viral entry into cells. Vero E6 cells were pretreated with 25-HC, and then infected with these luciferase-expressing pseudovirus. Intriguingly, we observed that cholesterol removal from plasma membranes failed to reduce pseudovirus infectivity (Figure 1D). Similarly, preincubation of pseudovirus with 25-HC at 12.5 μM also exhibited no significantly impact on the entry of virions (Figure 1E).

Our results showed that 25-HC, which lowers plasma membrane cholesterol, did not suppress the viral entry of SARS-CoV-2 and its variants, as well as SARS-CoV and MERS-CoV. However, 25-HC has been demonstrated to exhibit broad-spectrum antiviral activity against human coronaviruses (Wang et al., 2020; Zu et al., 2020; Lan et al., 2021). This apparent divergence may be attributed to the essential role of cholesterol-enriched host cell membrane in governing post-entry processes of enveloped virus, where they orchestrate the formation of viral replication compartments and subsequent assembly and budding of progeny virions (Li et al., 2020; Wudiri and Nicola, 2017; Osuna-Ramos et al., 2023; Hu et al., 2019). Consequently, treatment of host cells with 25-HC selectively blocks replication-competent authentic virus infection while demonstrating negligible effects on single-cycle pseudotyped particles.

### Cholesterol in the human coronavirus virions positively correlates with cellular infectivity

Given that 25-HC pretreatment of target cells lacks efficacy in virus entry models, we hypothesize that cholesterol content within the spike protein-containing viral membrane derived from the host cell may critically determine the entry efficiency of human coronaviruses. To validate this hypothesis, we applied methyl-*β*-cyclodextrin (MβCD), a compound that directly ‘strips’ membrane cholesterol without perturbing intracellular cholesterol homeostasis, to delineate the functional contribution of virion-associated envelope cholesterol to viral entry.

**Figure 2:**
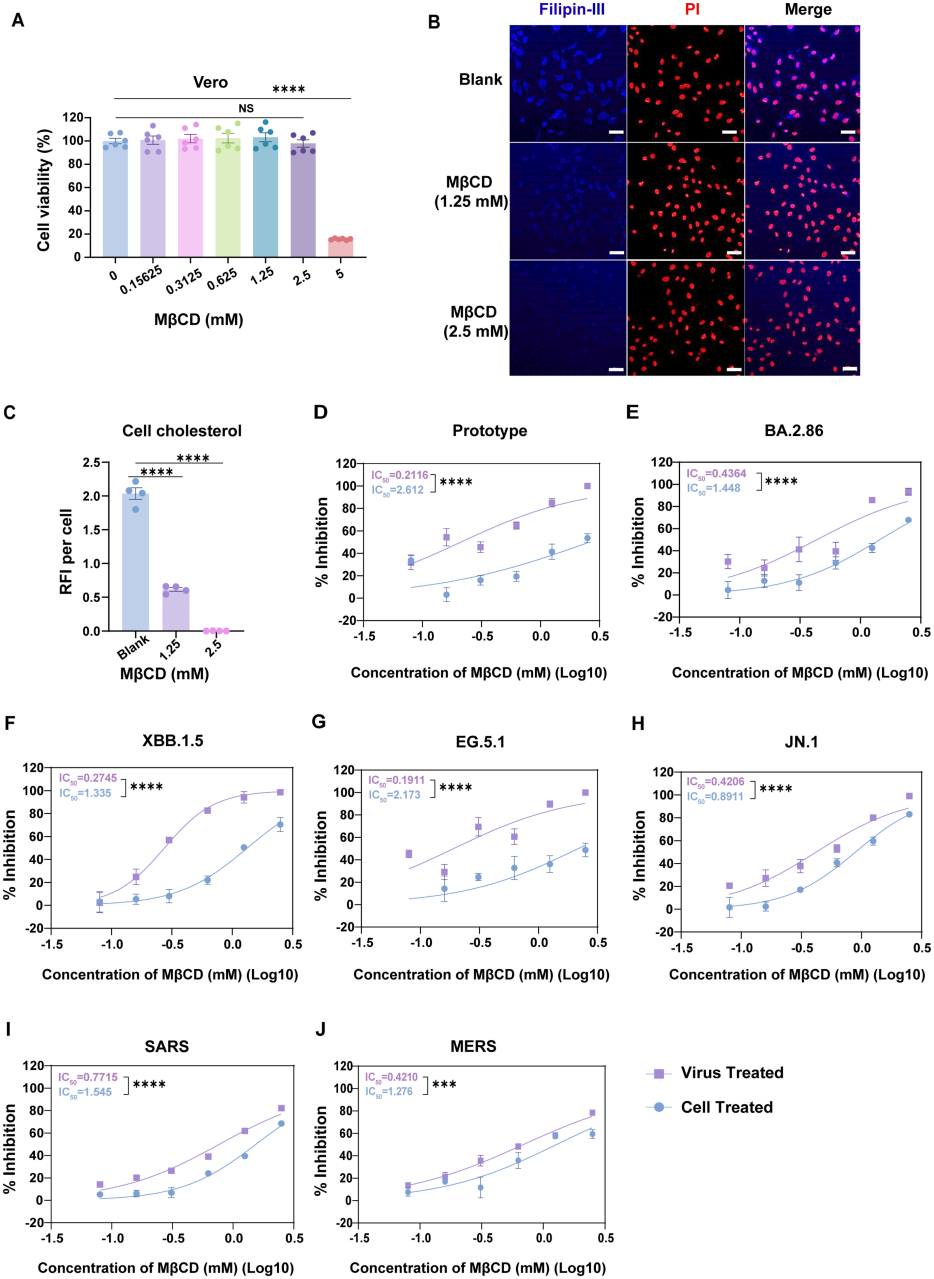
Cholesterol content within human coronavirus virions exhibits a positive correlation with viral infectivity. **(A)** Cytotoxicity of MβCD to Vero E6 cells was measured by CCK-8 assays. The *Y*-axis values of the graph represent the mean percentage of viable cells relative to untreated controls. **(B,C)** Immunofluorescence analysis of cholesterol levels in Vero E6 cells that were treated with the indicated concentrations of MβCD. Cholesterol was labeled with filipin-III, and the nuclei were stained with Propidium Iodide. The cholesterol distribution was measured by confocal microscopy and quantified by ImageJ. Scale bars, 50 μm. **(D–J)** Vero E6 cells or pseudovirus were pre-treated with serial dilutions of MβCD followed by PBS washes to remove residual MβCD prior to pseudovirus infection. Virus infection was quantified by luciferase assay at 24 h post-infection. The *Y*-axis of the graphs represent mean % inhibition of viral entry. IC_50_ was calculated by GraphPad using non-linear regression analysis. For all figures, experiments were repeated at least three times with similar results. Data are represented as mean ± SEM, *NS* not significant, *** *p* < 0.001, **** *p* < 0.0001. Statistical significance was determined by two-tailed unpaired Student’s *t* test for two groups or one-way ANOVA with Tukey’s HSD test for multiple groups.

MβCD treatment at 2.5 mM, the predetermined maximum non-cytotoxic concentration by CCK-8 assays (Figure 2A), induced near-complete membrane cholesterol depletion in Vero E6 cells, as quantified through filipin-III fluorescence intensity analysis (Figures 2B,C). We then directly incubated a concentration gradient of MβCD with SARS-CoV-2 pseudovirus encoding firefly luciferase prior to infection. Remarkably, MβCD treatment exhibited potent antiviral effect, achieving a half-maximal inhibitory concentration (IC_50_) of 0.21 mM (Figure 2D). Subsequent evaluation of SARS-CoV-2 variants revealed comparable antiviral efficacy with IC_50_ values below 0.5 mM (Figures 2E–H). Furthermore, similar inhibitory profiles were observed for SARS-CoV (IC_50_ = 0.77 mM) and MERS-CoV (IC_50_ = 0.42 mM), confirming broad-spectrum inhibitory effects (Figures 2I,J). Importantly, in these assays, the MβCD used to incubate with the virus was diluted out of samples upon infection, so residual MβCD would have minimal effects on cell-membrane cholesterol levels. In comparison, MβCD-induced cholesterol depletion in cellular membranes resulted in a marked decrease in antiviral activity, evidenced by > 10-fold elevation in IC_50_ for both SARS-CoV-2 prototype and EG.5.1 variant, and 2 ~ 3-fold IC_50_ increase in other variant strains, as well as SARS-CoV and MERS-CoV (Figures 2D–J).

**Figure 3:**
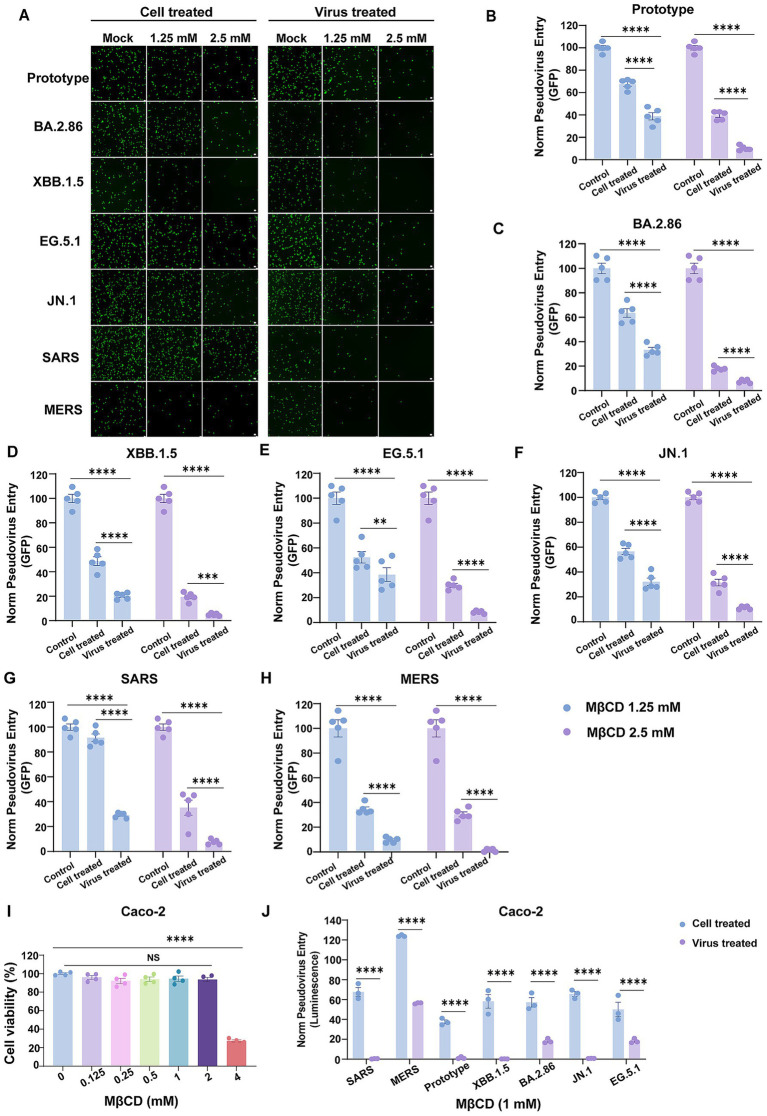
Viral envelope cholesterol plays a more critical role in mediating human coronaviruses’ cellular entry compared to host membrane cholesterol. **(A)** EGFP-encoding pseudoviruses of SARS-CoV-2, SARS-CoV, and MERS-CoV were pre-treated with indicated concentrations of MβCD before cellular infection, or alternatively, Vero E6 cells were incubated with MβCD for 2 h followed by PBS washes to remove residual MβCD prior to challenge by EGFP-encoding pseudovirus. Viral infections were visualized by fluorescence microscopy at 24 h post-infection. **(B–H)** Quantitation of data in panel **(A)**. Pseudovirus infection efficiency was calculated as the percentage of GFP-positive cells relative to mock-treated groups. **(I)** Cytotoxicity of MβCD to Caco-2 cells was measured by CCK-8 assays. The *Y*-axis values of the graph represent the mean percentage of viable cells relative to untreated controls. **(J)** Viral envelope cholesterol dependence in human coronaviruses’ cellular entry, regardless of the entry route supported by the target cell. Caco-2 cells or pseudovirus were treated and infected as in **(A)**. Virus infection was quantified by luciferase assay at 24 h post-infection. The *Y-*axis of the graphs represents the mean percentage of viral entry normalized to the control group. Data are represented as mean ± SEM, *NS* not significant, **p < 0.01, *** *p* < 0.001, **** *p* < 0.0001. Statistical significance was determined by two-tailed unpaired Student’s *t* test for two groups or one-way ANOVA with Tukey’s HSD test for multiple groups.

Parallel experiments employing EGFP-expression pseudovirus demonstrated comparable findings as shown by luciferase assays. Cholesterol extraction from pseudovirus using MβCD mediated nearly abolished viral infectivity ( > 90% suppression at 2.5 mM) (Figures 3A–H, right). Even at a lower concentration of 1.25 mM, we observed a ~ 60% reduction in GFP fluorescence (Figures 3B–H, left). Similarly, MβCD-mediated cholesterol depletion from Vero E6 membranes showed approximately 50% weaker inhibition of viral entry compared to viral envelope cholesterol modulation (Figures 3A–H).

Coronaviruses enter cells through two pathways: fusion at the plasma membrane or in the endosome in a cell-type-dependent manner (Zhu et al., 2021). We next investigated whether inhibition of viral entry via cholesterol depletion in the viral envelope exhibited cell-type specificity limited to Vero E6 cells or represented a broader cellular mechanism. To address this question, we quantified human coronaviruses entry into Caco-2 cells, which are primarily infected via the direct fusion pathway (Jackson et al., 2022). Concentrations of MβCD used in these experiments are non-cytotoxic (Figure 3I). We found that cholesterol depletion from viral envelopes completely blocked SARS-CoV entry into Caco-2 cells, while Caco-2 membrane cholesterol extraction achieved only partial inhibition (Figure 3J). These data, altogether, suggest that viral envelope cholesterol plays a more critical role in mediating human coronaviruses’ cellular entry compared to host membrane cholesterol, with virion-associated cholesterol levels acting as a key modulator of infectious potential regardless of the entry pathways.

### Validation of viral envelope cholesterol as a key factor in human coronavirus infection

**Figure 4:**
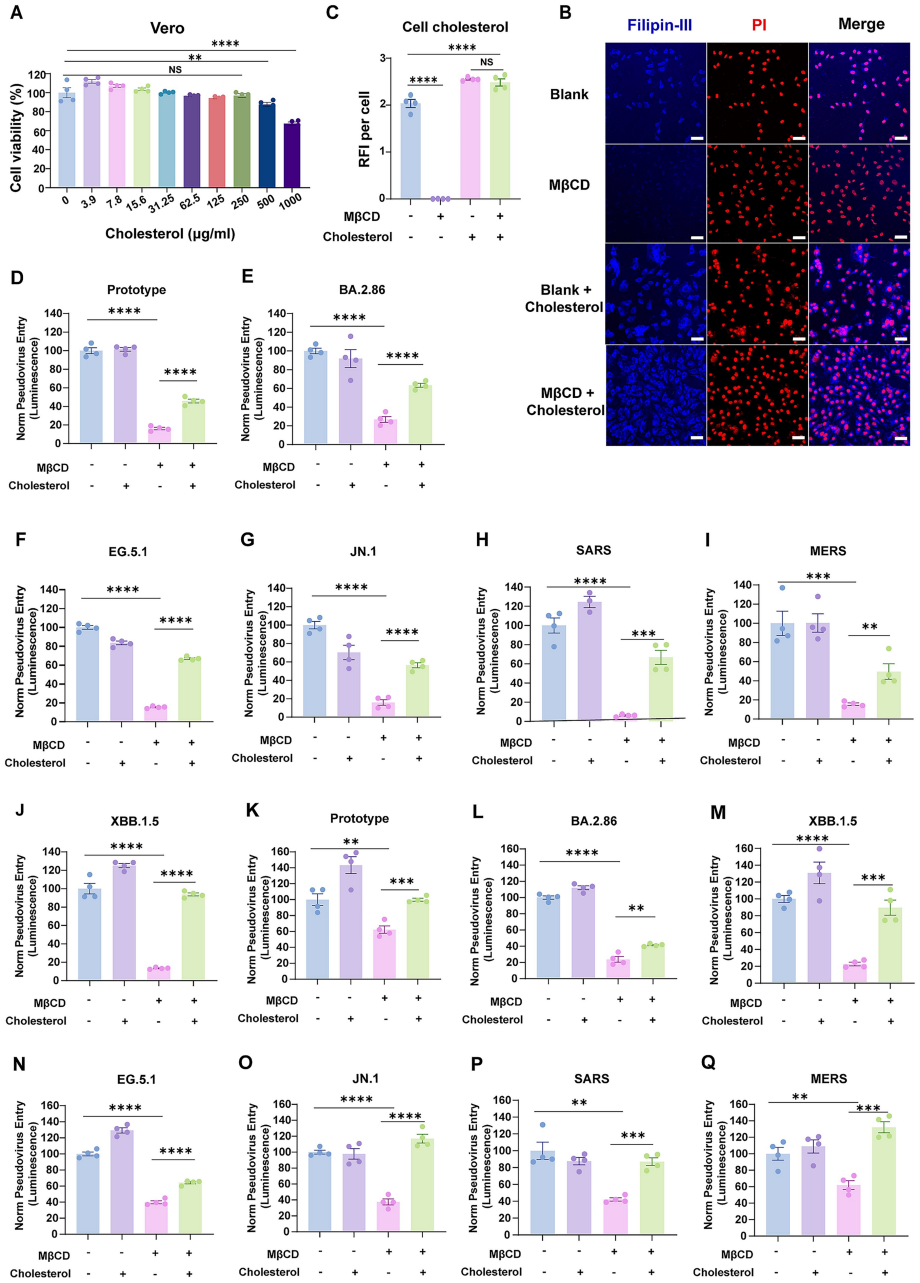
Cholesterol replenishment restored the infectivity of human coronaviruses with cholesterol-depleted envelopes. **(A)** Cytotoxicity of water-soluble cholesterol to Vero E6 cells was measured by CCK-8 assays. The *Y*-axis values of the graph represent the mean percentage of viable cells relative to untreated controls. **(B,C)** Immunofluorescence analysis of cholesterol levels in Vero E6 cells that were treated with 2.5 mM MβCD with or without water-soluble cholesterol. Cholesterol was labeled with filipin-III, and the nuclei were stained with Propidium Iodide. The cholesterol distribution was measured by confocal microscopy and quantified by ImageJ. Scale bars, 50 μm. **(D–J)** Pseudoviruses representing the SARS-CoV-2 prototype strain, Omicron variants, SARS-CoV, and MERS-CoV were treated with 2.5 mM MβCD in the presence or absence of water-soluble cholesterol before cellular infection. **(K–Q)** Vero E6 cells were treated with 2.5 mM MβCD with or without water-soluble cholesterol. Cells were washed twice and then infected with human coronaviruses. Virus infection was quantified by luciferase assay at 24 h post-infection. The *Y*-axis of the graphs represents the mean percentage of viral entry normalized to the control group. Data are represented as mean ± SEM, *NS* not significant, **p < 0.01, *** *p* < 0.001, **** *p* < 0.0001. Statistical significance was determined by two-tailed unpaired Student’s *t* test for two groups or one-way ANOVA with Tukey’s HSD test for multiple groups.

Theoretically, if MβCD-mediated cholesterol depletion within virions is essential for cellular entry, cholesterol replenishment should restore the impaired infectivity of MβCD-treated coronavirus. To this end, we performed a functional cholesterol rescue experiment to confirm the role for cholesterol within the virion membrane. We supplemented MβCD-treated cells with exogenous cholesterol in cyclodextrin complex at 250 μg/mL (Figure 4A), and found that the depletion of accessible cholesterol could be rescued by supplementing soluble cholesterol to the cell culture (Figures 4B,C).

SARS-CoV-2, SARS-CoV, and MERS-CoV pseudoviruses were first treated with MβCD, then assessed for cellular entry efficiency with or without a prior supplement of cholesterol. As expected, cholesterol supplementation restored entry capacity of envelope cholesterol-depleted pseudoviruses by up to 60% for the SARS-CoV-2 prototype strain, variants of BA.2.86/ EG.5.1/JN.1, SARS-CoV, and MERS-CoV (Figures 4D–I). Besides, restoration was achieved by up to 90% for XBB.1.5 variant (Figure 4J). Collectively, these findings supported the concept that virion membrane cholesterol content is responsible for coronavirus entry efficiency, and that the inhibitory effect of MβCD-mediated cholesterol depletion within virions exhibits partial reversibility upon cholesterol replenishment.

Given the dual inhibitory capacity of MβCD in depleting both virion-associated and host membrane cholesterol, we asked whether such inhibition was achieved through the same mechanism. To address this question, Vero E6 cells were pretreated with MβCD alone or subjected to cholesterol replenishment following MβCD treatment, and subsequently challenged with pseudoviruses. Remarkably, supplementation of cholesterol fully restored viral entry capacity across multiple tested strains, including SARS-CoV-2 (prototype, and XBB.1.5/JN.1 variants), SARS-CoV, and MERS-CoV (Figures 4K–Q). These data suggest that host cell plasma membrane cholesterol replenishment facilitated significantly greater recovery of viral entry efficiency compared to viral envelope cholesterol reconstitution. This functional disparity likely stems from the cholesterol-dependent structural integrity of S proteins in viral envelopes, where cholesterol depletion-induced conformational alterations exhibit irreversible characteristics that cholesterol reinsertion cannot fully rectify.

### Dynamic modulation of host cell membrane cholesterol directly governs progeny coronavirus virion infectivity

**Figure 5:**
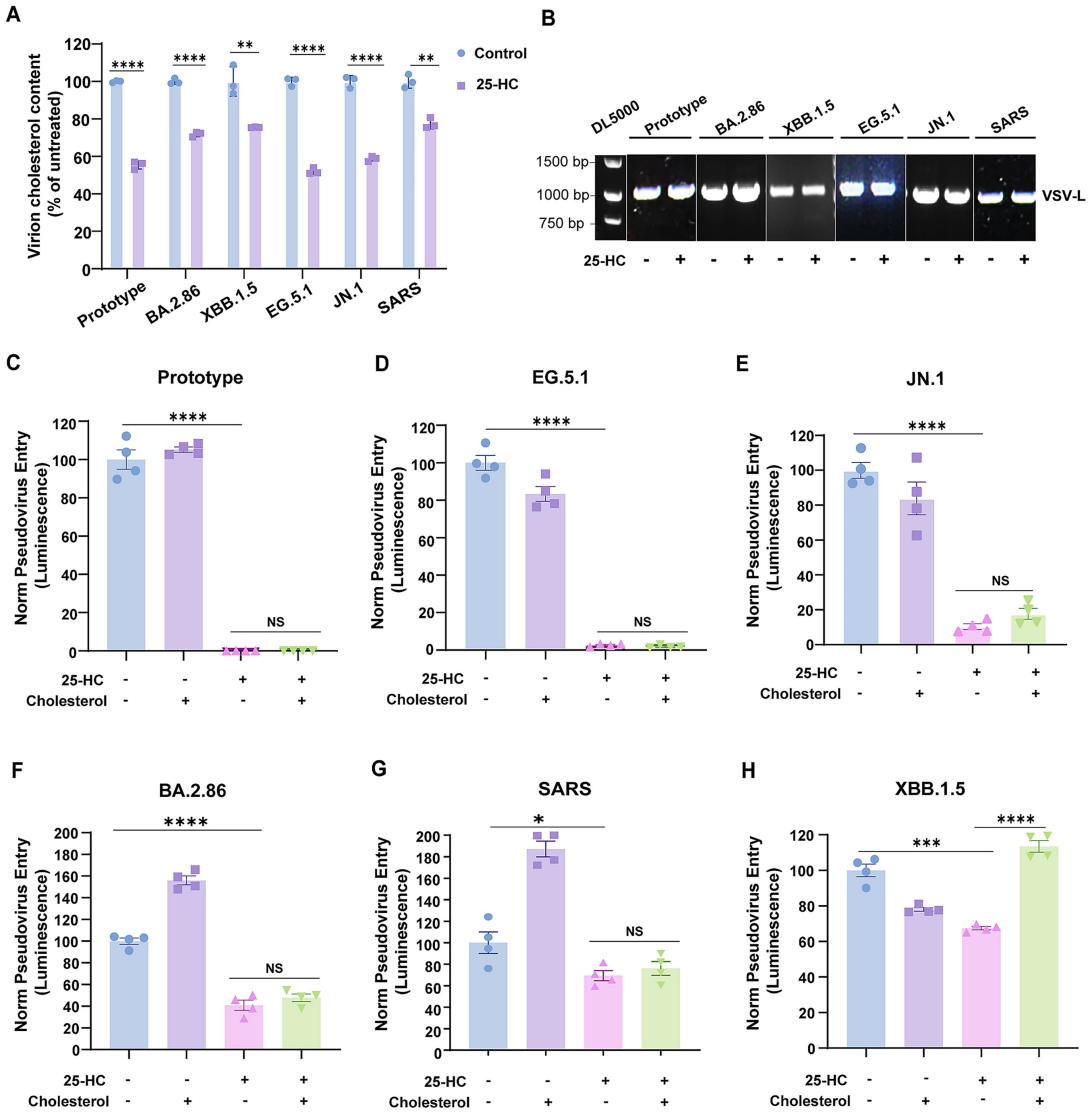
Exogenous cholesterol reconstitution failed to rescue the infectivity of coronaviruses generated in 25HC-treated cells. **(A)** The cholesterol content of virions produced in 25-HC-treated or mock-treated cells was measured using the Amplex Red Cholesterol Assay Kit. **(B)** Viral particles quantities were normalized through RT-PCR-based quantification of viral genomes using primers specific to the VSV-L gene segment. **(C–H)** Pseudoviruses representing SARS-CoV-2 prototype strain, Omicron variants, and SARS-CoV were produced in 25-HC-treated or mock-treated HEK293T cells, then added to Vero E6 cells with or without water-soluble cholesterol. Virus infection was quantified by luciferase assay at 24 h post-infection. The *Y*-axis of the graphs represents the mean percentage of viral entry normalized to the control group. Data are represented as mean ± SEM, *NS* not significant, *p < 0.05, **p < 0.01, *** *p* < 0.001, **** *p* < 0.0001. Statistical significance was determined by two-tailed unpaired Student’s *t* test for two groups or one-way ANOVA with Tukey’s HSD test for multiple groups.

To determine whether the impaired virus infection was indeed due to decreased cholesterol accumulation in the viral envelope, we subsequently characterized the infectivity of human coronavirus virions assembled under cholesterol-restricted host cell conditions. To this end, HEK293T cells expressing spike protein were pretreated with 25-HC to induce internalization of accessible cholesterol from the plasma membrane (Du et al., 2004), followed by G*ΔG-VSV infection, which generated progeny coronaviruses with reduced envelope cholesterol content (Figure 5A).

Virion titers were normalized by measuring viral genomes prior to conducting synchronized infection assays (Figure 5B). Our results demonstrated that SARS-CoV-2 prototype and EG.5.1 generated in 25-HC-treated HEK293T cells exhibited almost completely abolished entry capacity (Figures 5C,D). Quantitatively, JN.1 and BA.2.86 exhibited 80% and 50% reductions in entry efficiency, respectively, relative to untreated controls (Figures 5E,F). Furthermore, SARS-CoV pseudoviruses produced under the same conditions displayed a 30% reduction in infection rates (Figure 5G). Interesting, supplementing coronavirus virions produced in 25-HC-treated cells with exogenous cholesterol failed to restore infectivity. Of note, XBB.1.5 displays divergent characteristics relative to other SARS-CoV-2 variants, with cholesterol repletion rescuing its infectivity (Figure 5H). The mechanistic basis for these differences warrants future exploration. Collectively, these results establish viral envelope cholesterol as a critical determinant of human coronavirus infectivity, and suggest that cholesterol removal-induced viral envelope restructuring compromises envelope integrity, yielding permanent infection-incompetent virions. Our data supports a mechanistic model wherein 25-HC exerted its antiviral activity possibly by altering cholesterol levels in progeny virion envelopes.

### Cholesterol deficiency in human coronavirus envelopes impairs viral attachment to host cells

**Figure 6:**
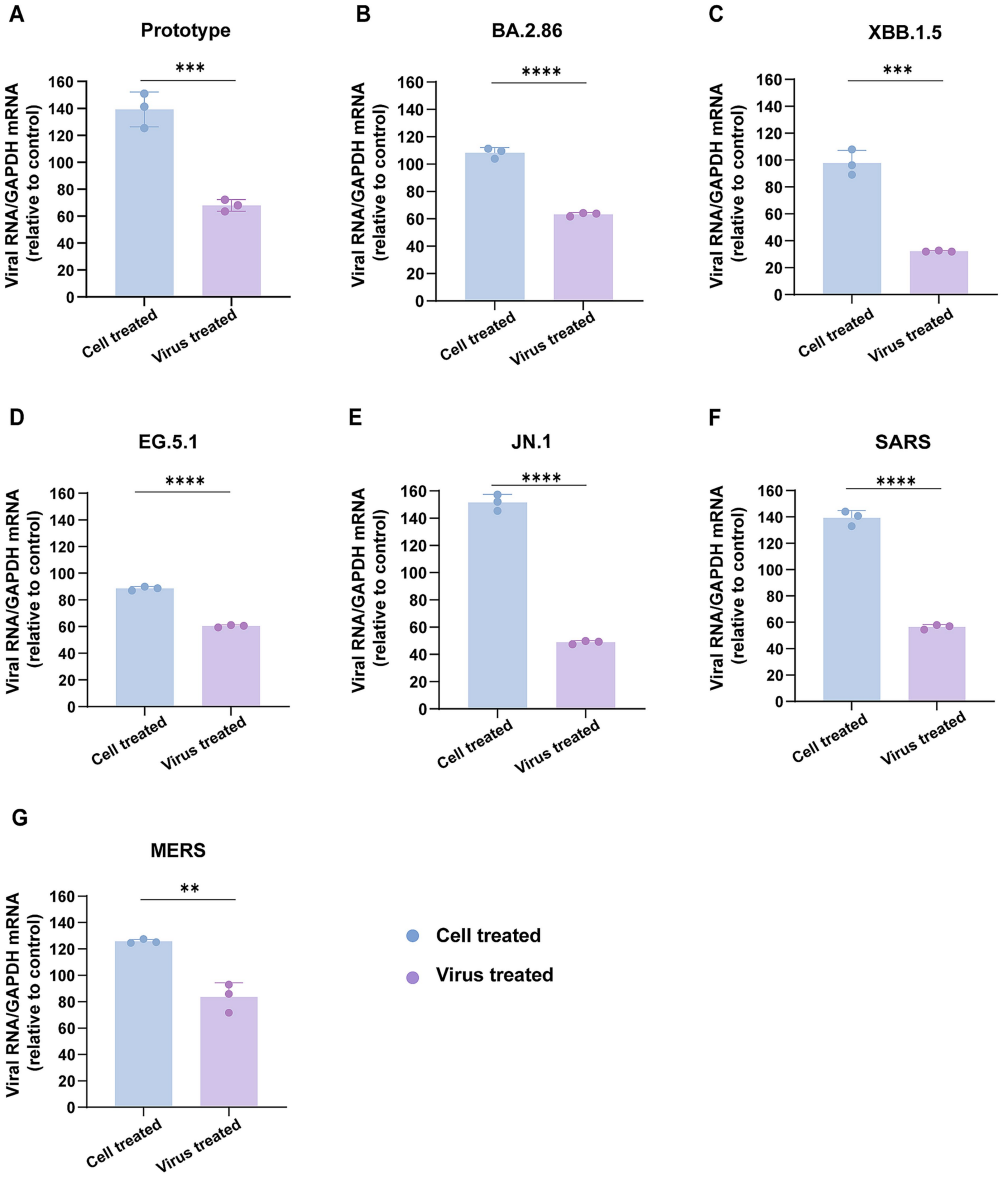
Lower cholesterol levels in coronavirus envelopes disrupt viral attachment to host cells. **(A–G)** MβCD-treated and untreated pseudoviruses were incubated with Vero E6 cells on ice for 30 min to assess cell surface attachment. Binding assays were also performed by treating Vero E6 cells with MβCD prior to pseudovirus challenge at 4 °C. RT-qPCR-based quantification of cell-associated viral genomes was employed to evaluate viral attachment capacity. The *Y*-axis of the graphs displays the mean relative abundance (%) of viral RNA, normalized to GAPDH and expressed relative to untreated controls. Data are represented as mean ± SEM, **p < 0.01, *** *p* < 0.001, **** *p* < 0.0001. Statistical significance was determined by two-tailed unpaired Student’s *t* test for two groups or one-way ANOVA with Tukey’s HSD test for multiple groups.

We next explored the mechanism by which envelope cholesterol depletion restricts coronavirus infection. We examined whether the envelope cholesterol was required for human coronavirus entry by allowing virus attachment to susceptible cells. Human coronavirus particles were pretreated with MβCD, followed by incubation with Vero E6 cells at 4 °C, a temperature condition that selectively permits viral attachment while blocking membrane fusion. Quantification of cell-associated viral genomes through RT–qPCR revealed a ~ 50% decrease in surface-bound viral RNA following MβCD-mediated cholesterol depletion, confirming viral envelope cholesterol-dependent binding impairment (Figures 6A–G). However, pretreatment of host cells with MβCD showed no significant effect on viral attachment efficiency, indicating MβCD blocks human coronavirus spike-mediated fusion with the plasma membrane, thereby suppressing viral entry (Figures 6A–G).

## Discussion

Enveloped viruses are highly dependent on their lipid envelopes for infection of host cells. SARS-CoV-2 assembles its host-derived lipid envelope through ERGIC-mediated budding, a process requiring cholesterol-rich microdomains. Our findings reveal that the envelope cholesterol of SARS-CoV-2 and related coronavirus is a critical factor that directly determines its infectivity. Depletion of cholesterol from the viral envelope significantly reduced virus infectivity, while cholesterol replenishment rescued viral entry efficiency. Parallel depletion in producer cells reduced virion cholesterol incorporation, yielding less infectious viral particles. Therefore, interfering with the viral lipid metabolism may reduce clinical complications in patients severely affected by COVID-19. Our data elucidate a potential viral strategy involving manipulating the envelope cholesterol to modulate host cell susceptibility among β-coronaviruses. Further structural and functional investigations are warranted to determine the optimal cholesterol proportion within the envelope required for viral infectivity.

We observed that 25-HC pretreatment of host cells did not significantly affect SARS-CoV-2 entry, whereas high-dose MβCD treatment induced moderate viral inhibition, suggesting that profound host membrane cholesterol removal is required to achieve significant viral entry inhibition. Notably, our results indicate that MβCD-mediated cholesterol depletion in virions reduced cellular infectivity by 2-fold compared to host membrane-treated controls, demonstrating the predominant role of the viral envelope cholesterol in the viral-host membrane binding. We speculate that the differential cholesterol requirement for viral entry observed between SARS-CoV-2 virions and host membranes may partly be due to their distinct membrane architectures, particularly divergent lipid-protein ratios, as well as membrane curvature. Indeed, the sensitivity of enveloped virus entry to viral envelope cholesterol levels is a ubiquitous feature, as observed in influenza A (Sun and Whittaker, 2003), HIV-1 (Graham et al., 2003), and Herpes simplex virus 1 (Wudiri et al., 2017). This conserved cholesterol-sensing mechanism reflects evolutionary selection for envelope membrane biophysical adaptations that enhance viral transmission efficiency.

It should be noted that the precise mechanisms connecting viral envelope cholesterol and SARS-CoV-2 infectivity remain unclear. Viral envelope cholesterol may facilitate SARS-CoV-2 cellular entry by modulating membrane fluidity, maintaining the quaternary structure of S protein trimers within virions, optimizing membrane curvature compatibility at the host-virus interface, and thermodynamically stabilizing fusion pore development (Mastrodomenico et al., 2022; Aliper et al., 2022; Meher et al., 2023; Lee et al., 2021). Additionally, SARS-CoV-2 has been shown to exploit cholesterol transport and synthesis in the infected cell to enhance its infectivity (Barrantes, 2022), thus we cannot exclude the possibility that viral envelope cholesterol may modulate viral infectivity beyond the entry step, such as through lipid-mediated cell signaling involved in immune evasion (Lim et al., 2022).

During the initial phase of the SARS-CoV-2 pandemic, epidemiological data revealed that SARS-CoV-2 exhibited distinct age-related progression to critical illness, while demonstrating particular predilection for patients with chronic diseases such as hypertension, diabetes, and cardiovascular diseases (Huang et al., 2021; Wu and McGoogan, 2020; Holly et al., 2020). The severity of disease following infection is determined by viral replication kinetics and infectious potential of progeny virions. Our proposed model postulates that age-related cholesterol accumulation in pulmonary cells potentiates nascent coronavirus virions infectivity via enhanced viral entry mediated by cholesterol-enriched viral envelope assembly in nascent virions, a mechanism similarly in patients with metabolic comorbidities. This model is supported by the findings that SARS-CoV-2 infection was greatly increased in APOE KO Huh7 cells, which exhibited increased cholesterol accumulation compared with WT cells (Gao et al., 2022). Altogether, our data provide partial mechanistic insights into prior studies demonstrating the pan-coronavirus inhibition potency of host cholesterol-modulating interventions (Schneider et al., 2021; Wing et al., 2023). Future studies should delineate whether atherosclerotic plaque-associated arterial microenvironments potentiate the packaging of SARS-CoV-2 virions with enhanced infectivity profiles compared to non-atherosclerotic vascular segments.

The normal lipid metabolism in the host cell is altered as a consequence of viral infections (Wudiri and Nicola, 2017; Sun and Whittaker, 2003; Contreras et al., 2021). It has been indicated that the lipidomic profiles of the envelope play a critical role in the viral infectivity, with spatiotemporal sorting of specific lipid species occurring during enveloped virion morphogenesis (Callens et al., 2016; Brügger et al., 2006; Alketbi et al., 2021). In addition to cholesterol, the functional roles of other lipid components in the viral envelope and their contributions to viral pathogenesis also require further elucidation.

A limitation of this study is the exclusive reliance on VSV pseudovirus systems throughout our experimental investigations. While this approach provides a valuable and safe model for studying specific aspects of the viral entry process, they inherently lack the full biological complexity of authentic, replication-competent live viruses. Future validation using authentic live virus assays under appropriate biosafety containment is essential to confirm the biological relevance and translational potential of these observations. Furthermore, *in vivo* studies utilizing animal models of coronavirus infection and pathogenesis are critically required to characterize the therapeutic potential and physiological impact of the antiviral compounds targeting the viral lipidome.

SARS-CoV-2 variants of concern (VOC) are emerging and spreading, SARS-CoV-2 pandemic is likely to remain a global health threat for the foreseeable future. Our data support synergistic drug combinations of viral lipid-targeting antivirals in conjunction with the inhibitors targeting the intracellular stages of the SARS-CoV-2 replication cycle, such as 3CLpro (main protease) or RdRp (RNA-dependent RNA polymerase) (Xu et al., 2022).

## Materials and methods

### Cell culture

Embryonic kidney cell lines (HEK293T), human colon epithelia cell line (Caco-2), African Green Monkey Kidney Cell (Vero E6) were purchased from ATCC. HEK293T and Vero E6 cells were grown in DMEM medium and Caco-2 cells were cultured in MEM medium. All the medium was supplemented with 10% fetal calf serum, 100 U/mL penicillin, and 100 μg/mL streptomycin, and maintained at 37 °C in a 5% CO_2_ incubator.

### Reagents

Luciferase assay kit was purchased from Vazyme (DD1204-03). 25-Hydroxycholesterol (H1015), Methyl-*β*-Cyclodextrin (C4555), and water-soluble cholesterol (01692183) were obtained from Sigma-Aldrich. Transfection reagent (PEI) was purchased from LABLEAD (P4000). Amplex Red Cholesterol Assay Kit (S0211S) was purchased from Beyotime.

### Cytotoxicity assay

The cytotoxicity of the tested drugs on Vero E6 or Caco-2 cells were determined by Cell Counting Kit-8 (CCK-8) assays according to the manufacturer’s instructions. Briefly, cells were seeded into 96-well plates and incubated with different compounds, including 25-HC, MβCD, or water-soluble cholesterol at the indicated concentrations in triplicate. After 24 h incubation at 37 °C, the CCK-8 cell viability reagent was added directly to cells and incubated for a further 1 h at 37 °C. The absorbance of each well at 450 nm was recorded, and the cytotoxic concentration was calculated after adjusting the absorbance for background and comparing to untreated controls.

### Production and titration of pseudotyped virus

Pseudotyped viruses for the SARS-CoV-2 prototype strain, its variants, as well as MERS and SARS coronaviruses were generated with vesicular stomatitis virus (VSV) pseudotyping system (Nie et al., 2020). In detail, plasmids encoding spike protein were transfected into HEK293T cells to provide membrane proteins on the surface of cells. VSV G pseudotyped virus (G*ΔG-VSV) were added 24 h after the transfection and removed after 2 h incubation. Viral supernatants were harvested after another 30 h, passed through a 0.45 μm filter, aliquoted, and stored at −80 °C. The 50% tissue culture infectious dose (TCID_50_) was calculated using the Reed–Muench method (Matumoto, 1949).

### Confocal ‌ imaging of cholesterol

Cells grown on poly-L-lysine-coated glass coverslips were treated with the indicated concentrations of 25-HC or MβCD. Cells were fixed in 4% paraformaldehyde for 10 min, permeabilized with 0.3% Triton X-100 for 10 min, and finally blocked by 2% BSA in PBS for 1 h. Cells were incubated with filipin-III in the dark for 1 h and washed three times with PBS. Nuclear DNA staining was performed with Propidium Iodide (MCE, HY-D0815) according to the manufacturer’s instructions. The coverslips were mounted with mounting medium and then imaged by confocal microscopy. The Propidium Iodide (PI) channel was used to count the number of cells per image. Fluorescence was divided by cell count to calculate the fluorescence per cell in each condition.

### Pseudovirus entry assay

Pseudotyped virus containing spike proteins of human coronavirus were incubated with specified concentrations of 25-HC or MβCD at 37 °C for 2 h. Then, the treated virus was diluted in serum-free DMEM and applied to pre-seeded cell monolayers with or without water-soluble cholesterol (250 μg/mL). Following 2 h adsorption at 37 °C, the inoculum was replaced with maintenance medium (DMEM supplemented with 2% FBS). For pseudovirus with Fluc, luciferase activity in cell lysates at 24 h post-infection was determined and compared with the control groups. For EGFP pseudovirus, infected was evaluated by taking pictures with the inverted fluorescence microscope for at least 5 random fields at 24 h post-infection, and the percentage of positive cells was calculated.

### Production and quantification of Pseudotyped viruses with reduced envelope cholesterol content‌

For plasma membrane cholesterol depletion, HEK293T cells were treated with 25-HC (12.5 μM) for 24 h prior to transfection with plasmids encoding variant spike protein. Cells were infected with VSV G pseudotyped virus (G*ΔG-VSV) at 24 h post-transfection. The infection medium was removed after 2 h incubation, and was changed into fresh DMEM with 2% FBS. The supernatants containing pseudovirus were collected, centrifuged, and stored at −80 °C after another 24 h incubation. RT-PCR was performed to quantify viral genome copies, thereby estimating virus particle counts. The cholesterol content of produced virions was determined using an Amplex Red Cholesterol Assay Kit, according to the manufacturer’s instructions.

### Viral attachment assay

Vero E6 cells were seeded in 12-well plate one day prior to the assays. For virus attachment, cell culture plates were pre-chilled on ice for 10 min, followed by incubation with either MβCD-treated or mock-treated viral particles at 4 °C for 30 min. Cells were then washed with chilled PBS three times to remove unbound virions and lysed with TRIzol. The amounts of bound virions were quantified by analyzing cell-associated viral RNA through RT-qPCR using primers for VSV-L.

### RNA extract and RT-qPCR

Total RNA was extracted by RNAiso Plus (TaKaRa, 9,109); the RNA was then reverse-transcribed into cDNA with reverse transcription kit (TaKaRa, RR047A) according to the manufacturer’s instructions. qPCR was performed with technical duplicates using VSV-L gene-specific primers and TB Green Real Time PCR Kit (TaKaRa, RR820A). Delta cycle thresholds were calculated using GAPDH as the endogenous housekeeping gene control. Primers against the VSV-L genome were 5′-GACGGGCTCATCAGTCTATTT-3′(forward) and 5′-GGATACCTCACTCCTCACAATC-3′ (reverse). Primers against the GAPDH genome were 5’-AACGGATTTGGTCGTATTGGG-3′(forward) and 5’-TCGCTCCTGGAAGATGGTGAT-3′(reverse).

### Statistical analysis

All experiments were repeated at least three times with similar results. Statistical analysis was performed using GraphPad Prism 9. Statistical significance was assessed using two-tailed unpaired Student’s t-tests for comparisons between two groups. For analyses involving three or more groups, significance was calculated using an ordinary one-way ANOVA with Tukey’s Honestly Significant Difference (HSD) test. *p* values < 0.05 are considered significant and denoted as *p < 0.05, **p < 0.01, ***p < 0.001, and ****p < 0.0001. Nonsignificant values are denoted as ns.

## Data Availability

The original contributions presented in the study are included in the article/supplementary material, further inquiries can be directed to the corresponding authors.
